# Vasopressin for persistent hypotension due to amlodipine and olmesartan overdose: A case report

**DOI:** 10.1016/j.amsu.2021.102292

**Published:** 2021-04-15

**Authors:** Susumu Matsushime, Akira Kuriyama

**Affiliations:** Emergency and Critical Care Center, Kurashiki Central Hospital, 1-1-1 Miwa Kurashiki Okayama, 710-8602, Japan

**Keywords:** Angiotensin receptor antagonists, Drug overdose, Vasopressins, CCB, calcium channel blocker, ARB, angiotensin II receptor blocker, ED, emergency department, ACE, angiotensin-converting enzyme

## Abstract

**Background:**

While there are consensus recommendations for managing calcium channel blocker (CCB) toxicity, reports on angiotensin II receptor blocker (ARB) toxicity and management are limited. Herein, we report a case of catecholamine-refractory hypotension due to CCB and ARB overdose.

**Case presentation:**

A 54-year-old woman with underlying hypertension was brought to the emergency department after she attempted suicide by ingesting 345 mg of amlodipine, a CCB, and 340 mg of olmesartan, an ARB. She was hypotensive, which was considered vasodilatory because of high cardiac and low systemic vascular resistance indices. Hypotension persisted despite the administration of norepinephrine and epinephrine. Intravenous calcium gluconate, glucagon, and high-dose insulin euglycemia therapy, which were initiated because CCB toxicity was suspected, failed to raise her blood pressure. The presence of normal anion-gap metabolic acidosis and the fact that the patient remained hypotensive suggested that the hypotension might have been due to the effect of ARB. Vasopressin was finally administered, which improved her hemodynamic status. She was weaned off all vasopressors on day 3.

**Discussion:**

There is no consensus recommendation for ARB toxicity. Since chronic use of ARBs at conventional doses can block the sympathetic nervous and renin–angiotensin systems, catecholamines may not effectively increase blood pressure in cases of hypotension due to ARB overdose, for which vasopressin could be indicated.

**Conclusions:**

Vasopressin could be an option for treating hypotension secondary to ARB and CCB toxicity when catecholamines and treatment for CCB toxicity fail.

## Highlights

• Chronic use of ARBs blocks the sympathetic nervous and renin–angiotensin systems.
• There is no consensus recommendation for angiotensin II receptor blocker (ARB) toxicity.
• Catecholamines may not effectively raise blood pressure in hypotension due to ARB toxicity.
• Vasopressin could be an option for treating hypotension secondary to ARB toxicity.

## 1 Introduction

There are consensus recommendations for managing calcium channel blocker (CCB) toxicity, and several treatment options for CCB toxicity, including intravenous calcium, high-dose insulin euglycemia therapy (HIET), lipid emulsion therapy, and glucagon, are recommended [1]. In contrast, reports on angiotensin II receptor blocker (ARB) toxicity and management are limited. Further, a previous study suggested that ARB toxicity was associated with no or minor symptoms [2].

We report a patient with amlodipine and olmesartan overdose who developed prolonged hypotension refractory to catecholamines. Vasopressin successfully increased the blood pressure of our patient. This case was reported according to SCARE statement [3].

## 2 Case Presentation

A 54-year-old woman with a hypertension and insomnia history visited our emergency department (ED) with acute altered mental status. She had ingested amlodipine 345 mg, olmesartan 340 mg, etizolam 4 mg, and brotizolam 0.75 mg approximately 10 h before arrival, because she felt exhausted by caring for her family. The patient had been prescribed amlodipine 5 mg and olmesartan 20 mg daily. Thus, she had ingested 69- and 17-fold her usual daily dose of amlodipine and olmesartan, respectively. She had attempted suicide by overdose previously. Her height and weight were 158 cm and 77.5 kg, respectively. She presented with a blood pressure of 84/46 (mean arterial pressure: 59) mmHg, heart rate of 72 bpm, respiratory rate of 22 bpm, SpO_2_ of 98% on a 5 L/min facemask, and temperature of 35.3 °C. Her Glasgow Coma Scale score was 9 (E2V1M6); physical examination results were normal except for cold extremities. Laboratory examinations showed a serum creatinine level of 1.06 mg/dl and glucose level of 156 mg/dl; other chemistry panel results were within normal limits. Whole-body computed tomography showed no signs of infections. Transthoracic echocardiography showed normal contractility (left ventricular fraction ejection: 50%).

Despite crystalloid fluid (1500 mL) administration, the patient remained hypotensive and norepinephrine treatment was initiated after intensive care unit admission. However, her mental status deteriorated, necessitating intubation. Her hemodynamic status was refractory to increased norepinephrine; therefore, epinephrine was administered. CCB toxicity was suspected; hence, intravenous calcium gluconate (10.2 g in total) and glucagon (1 mg in total) were repeatedly administered. However, vasoplegia persisted at 8 h after ED arrival. Once norepinephrine and epinephrine doses reached 0.45 and 0.19 mcg·kg^−1^·min^−1^, respectively, we added intravenous insulin (70 U/h) as high-dose insulin euglycemia therapy. Nevertheless, she remained hemodynamically unstable (maximum mean arterial pressure: 56 mmHg). Arterial blood gas analysis showed normal anion-gap metabolic acidosis (pH: 7.127; PaCO_2_: 45.8 mmHg; HCO_3_: 14.5 mmol/L; lactate level: 9.2 mmol/L; and corrected anion gap: 12.5 mmol/L). FloTrac sensor (Edwards Lifesciences, Irvine, CA, USA) showed that her cardiac and systemic vascular resistance indices were 3.2 L·min^−1^·m^−2^ and 1015 dyne·s·cm^−5^·m^2^, respectively, indicating vasodilatory shock. The vasodilatory shock was refractory to CCB toxicity treatments; therefore, an ARB adverse effect was suspected. Vasopressin (maximum: 2 U/h) was administered at 16 h after ED arrival, which increased the mean arterial pressure to >60 mmHg (Figure). As the patient's blood pressure started to increase on hospital day 2, vasopressors were tapered and she was weaned off on hospital day 3. She was thereafter extubated and discharged without sequelae on hospital day 9. She no longer had suicidal ideation as of the discharge. She had an uneventful clinical course during the 2-month outpatient follow-up and remained well after 22 months of this event.

**Fig. 1:**
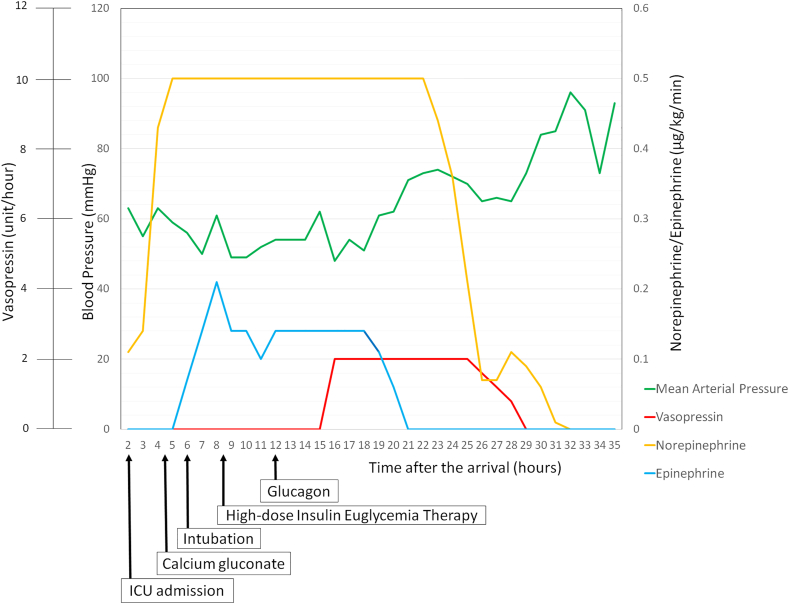
Changes in blood pressure associated with vasopressors.

## 3 Discussion

Amlodipine is a vascular-selective, long-acting CCB, with indications including hypertension and ischemic heart disease. It is reported to inhibit angiotensin-converting enzyme (ACE) in rabbits and in vitro [4]. CCBs block calcium entry into vascular smooth muscles and reduce peripheral vascular resistance. Olmesartan is an ARB mainly indicated for hypertension. ARBs block AT_1_ receptors, thereby reducing vasoconstriction, aldosterone release, and sympathetic nerve activity. Olmesartan is eliminated by hepatic metabolism and its half-life is 10–15 h [5].

Previous case reports suggested that the main manifestations of amlodipine toxicity include hypotension and hyperglycemia. There are multiple treatment choices for CCB toxicity [1]. Intravenous calcium administration increases extracellular calcium concentration and improves contractility and blood pressure. HIET is indicated for cardiogenic shock in CCB toxicity because it allows myocardial cells to uptake glucose, while insulin has concentration-dependent inotropic effects on myocardial cells. Lipid emulsion therapy has four mechanisms: reversal of sodium channel blockade, cytoprotective effect, inotropic effect, and metabolism facilitation. Glucagon has positive inotropic and chronotropic effects on the heart muscle [6]. According to the expert consensus recommendations, HIET is indicated as a first-line therapy for symptomatic CCB-poisoned patients with or without myocardial dysfunction and is intensified if the patients are refractory to the first-line therapy and develop cardiogenic shock. Lipid emulsion therapy is not the first-line therapy for CCB toxicity and is indicated when the patient is refractory to the first-line therapy or in the periarrest period. The consensus recommendations do not suggest the use of glucagon because vomiting and hyperglycemia have been reported in a case series.

In our patient, we did not intensify HIET and glucagon, because the hypotension was mainly vasodilatory and not due to cardiogenic shock. We also did not initiate lipid emulsion therapy because the patient had shock that was vasodilatory and not cardiogenic and or was not periarrest. Methylene blue is anecdotally used for CCB toxicity, but we did not administer it to our patient because the expert consensus recommendations were against its use for CCB toxicity given experience is limited to a few case reports, and because it is only indicated for methemoglobinemia in Japan. Normal anion-gap metabolic acidosis and lack of response to CCB toxicity treatments suggested lingering effects of the ARB in our patient.

A case series suggested ARB toxicity was associated with no or minor symptoms [2]. However, few case reports have described ARB toxicity–associated life-threatening hypotension. McNamee et al. reported that norepinephrine and phenylephrine failed to increase blood pressure in a 54-year-old woman with irbesartan overdose [7]. Jouffroy et al. reported that a 59-year-old man went into cardiac arrest after consuming excessive doses of antihypertensive drugs (irbesartan and lercanidipine) and anti-arrhythmic drugs [8]. Despite extracorporeal life support and norepinephrine and epinephrine administration, hypotension persisted. Thus, refractory and persistent hypotension can develop in cases of ARB toxicity.

In the aforementioned cases, terlipressin, a vasopressin analog, raised the blood pressure and reduced the requirement of other vasopressors. Terlipressin and vasopressin stimulate the vascular V1a receptors, leading to vasoconstriction. To our knowledge, vasopressin has rarely been considered an option for increasing blood pressure in a patient with ARB overdose or toxicity. A recent randomized trial suggested that angiotensin II effectively increased blood pressure in patients with refractory shock; however, it is not readily available [9]. Thus, currently, vasopressin can be a possible treatment option for hypotension due to ARB toxicity refractory to conventional vasopressors such as norepinephrine or epinephrine. Vasopressin could be titrated up to 0.04 U/min (equivalent to 2.4 units/h), because higher doses are associated with more side effects [10]. Thus, we decided to administer a dose of up to 2 units/h in our patient.

ARBs may inhibit central and peripheral sympathetic nerve activity [11], and patients chronically receiving ARBs or ACE inhibitors are prone to hypotension [12]. ACE inhibitors and ARBs also prevent vasopressin-mediated responses. A randomized trial suggested that compared with norepinephrine, vasopressin afforded a rapid reversal of hypotension after general anesthesia induction in patients chronically receiving renin–angiotensin system inhibitors [13]. Because chronic use of ARBs at conventional doses can block the sympathetic nervous and renin–angiotensin systems, catecholamines may not effectively increase blood pressure in cases of hypotension due to ARB overdose. Although it was unclear whether our patient had olmesartan toxicity, hypotension might have been augmented by the direct effect of and adverse effect due to chronic use of olmesartan in addition to effects of amlodipine.

## 4 Conclusion

There is no consensus recommendation for ARB toxicity. Vasopressin could be an option for treating hypotension secondary to ARB and CCB toxicity when catecholamines and treatment for CCB toxicity fail.

## Sources of funding

There are no sources of funding.

## Ethical approval

Not required for case reports.

## Informed consent

Written informed consent was obtained from the patient for publication of this case report and accompanying images. A copy of the written consent is available for review by the Editor-in-Chief of this journal on request.

## Author contributions

SM and AK looked after the patient, wrote and revised the draft, and approved the submission the current article.

## Research registration

Not applicable.

## Provenance and peer review

Not commissioned; externally peer-reviewed.

## Funding

None.

## Declaration of competing interest

The authors declare that they have no conflict of interest.

## References

[1] Agha R.A., Franchi T., Sohrabi C., Mathew G., Kerwan A., Group S. (2020). The SCARE 2020 guideline: updating consensus surgical CAse REport (SCARE) guidelines. Int. J. Surg..

[2] Xu B., Xiao-hong L., Lin G., Queen L., Ferro A. (2002). Amlodipine, but not verapamil or nifedipine, dilates rabbit femoral artery largely through a nitric oxide- and kinin-dependent mechanism. Br. J. Pharmacol..

[3] Schwocho L.R., Masonson H.N. (2001). Pharmacokinetics of CS-866, a new angiotensin II receptor blocker, in healthy subjects. J. Clin. Pharmacol..

[4] St-Onge M., Anseeuw K., Cantrell F.L., Gilchrist I.C., Hantson P., Bailey B. (2017). Experts consensus recommendations for the management of calcium channel blocker poisoning in adults. Crit. Care Med..

[5] Graudins A., Lee H.M., Druda D. (2016). Calcium channel antagonist and beta-blocker overdose: antidotes and adjunct therapies. Br. J. Clin. Pharmacol..

[6] Prasa D., Hoffmann-Walbeck P., Barth S., Stedtler U., Ceschi A., Farber E. (2013). Angiotensin II antagonists - an assessment of their acute toxicity. Clin. Toxicol..

[7] McNamee J.J., Trainor D., Michalek P. (2006). Terlipressin for refractory hypotension following angiotensin-II receptor antagonist overdose. Anaesthesia.

[8] Jouffroy R., Pegat-Toquet A., Bourdiault A., Philippe P., Carli P. (2018). Refractory vasodilatory shock induced by Irbesartan's acute intoxication. Clin Med Rev Case Rep.

[9] Khanna A., English S.W., Wang X.S., Ham K., Tumlin J., Szerlip H. (2017). Angiotensin II for the treatment of vasodilatory shock. N. Engl. J. Med..

[10] den Ouden D.T., Meinders A.E. (2005). Vasopressin: physiology and clinical use in patients with vasodilatory shock: a review. Neth. J. Med..

[11] Ye S., Zhong H., Duong V.N., Campese V.M. (2002). Losartan reduces central and peripheral sympathetic nerve activity in a rat model of neurogenic hypertension. Hypertension.

[12] Eyraud D., Brabant S., Nathalie D., Fleron M.H., Gilles G., Bertrand M. (1999). Treatment of intraoperative refractory hypotension with terlipressin in patients chronically treated with an antagonist of the renin-angiotensin system. Anesth. Analg..

[13] Boccara G., Ouattara A., Godet G., Dufresne E., Bertrand M., Riou B. (2003). Terlipressin versus norepinephrine to correct refractory arterial hypotension after general anesthesia in patients chronically treated with renin-angiotensin system inhibitors. Anesthesiology.

